# An overview of the commercial determinants of health

**DOI:** 10.1186/s12992-020-00607-x

**Published:** 2020-08-17

**Authors:** Melissa Mialon

**Affiliations:** School of Public Health, University of São Paulo, São Paulo, Brazil

**Keywords:** Commercial determinants of health, Corporate political activity, Industry, Public health, Non-communicable diseases

## Abstract

**Background:**

Different terms are described in the literature that refer to commercial determinants as drivers of ill-health. The aim of the present review was to provide an overview of the commercial determinants of health, through a review of the literature on this subject. The review was conducted in December 2019 and updated in February 2020. Searches were conducted from peer-reviewed scientific articles, commentaries, books, and books chapters, with no restriction in their publication dates and languages.

**Main body:**

The commercial determinants of health cover three areas. First, they relate to unhealthy commodities that are contributing to ill-health. Secondly, they include business, market and political practices that are harmful to health and used to sell these commodities and secure a favourable policy environment. Finally, they include the global drivers of ill-health, such as market-driven economies and globalisation, that have facilitated the use of such harmful practices.

**Short conclusion:**

The discussion on the commercial determinants of health offers a unique opportunity to shift the dominant paradigm in public health, where individual behaviours are considered to be driven by inadequate environments. Ill-health, damages to the environment, and health and social inequalities, might be better understood through a commercial determinant lens.

## Background

There is an emergent discussion, at the international level, on the commercial determinants as drivers of ill-health [1–3], amongst other social determinants [4]. Different terms are described in the literature to refer to these determinants. In 2013, Millar introduced the term ‘corporate determinants of health’ to refer to both the positive and negative influence that corporations have on population health, an idea further developed by other academics, such as Rochford et al. [5, 6]. Millar argued that companies generate employment and are a source of revenue through taxes, thus supporting the economic development of countries [5]. The author refers to the triple bottom line of corporations: people, planet and profits [5]. Some scholars have, however, argued that corporations’ search for profit is in direct contradiction with public health goals [7]. In his work, Millar acknowledges that, in some cases, corporations have a negative impact on health and the environment [5]. The term ‘commercial determinants of health’ (CDoH) was first introduced in the literature in 2013 by West and Marteau [8]. The term became popular after 2016 when used by Kickbush et al. define CDoH as “strategies and approaches used by the private sector to promote products and choices that are detrimental to health” [1]. Other terms have also been used in the literature: the ‘commercial determinants of ill health’ [9]; the ‘commercial drivers of ill-health’ [10]; the ‘commercial determinants of non-communicable diseases’ (NCDs) [10]; the ‘commercial drivers of NCDs’ [1]. The World Obesity Federation, a global professional association, uses the term ‘commercial determinants of obesity’ [11].

Some have described the development of conceptualizing CDoH, as a separate object of inquiry, over time, and proposed potential solutions to address one of the most pressing challenges in public health [2]. Others have developed models to understand [1] and study CDoH [12, 13]. Maani et al. recently noted that there is, however, a lack of clarity on what the term CDoH means [14]. In another publication, Maani et al. noted that commercial determinants remain largely ignored in existing frameworks on the social determinants of health [4]. The aim of the present review was to provide an overview of CDoH and feel the gap on the understanding of CDoH.

## Methods

The review was conducted in December 2019 and updated in February 2020. The author identified relevant scientific literature, including peer-reviewed articles, commentaries, books, and books chapters with no restriction in their publication dates and languages. Searches were initially conducted using the bibliography section in Kickbush et al.’s article [1] for publications that focused on CDoH, industrial epidemic, or corporate political activity (CPA). Forward (i.e., citations referring to this initial set of documents, using Scopus) and backward searches (i.e., references section of these documents) were then conducted using these publications. Additional documents discussing specific aspects of CDoH were then identified through snowball searches. All documents identified for this review were managed with F1000 Workspace.

The term ‘corporate’, used for example by Millar, usually refers to an organisation, while the term ‘commercial’ relates to activities intended to make a profit. The present review used the term ‘commercial determinants of health’ and was guided by the above definition by Kickbush et al. For the present review, the term ‘corporations’ refers to the individuals and organisations involved in the production, distribution and marketing of commodities: manufacturers, wholesalers, retailers, distributors, service providers, and producers of raw material, as well as organisations acting on their behalf, such as trade associations, public relations firms, philanthropic organisations, and research institutions. Freudenberg described these individuals and organisations as the ‘corporate consumption complex’ [15].

Kickbush et al.’s framework was used to structure the present review. This framework first presents the outcomes resulting from CDoH, including ill-health [1]. The authors explain that different channels, such as marketing and lobbying, described in this review as ‘corporate practices harmful to health’, are driving these outcomes [1]. Finally, Kickbush et al. suggest that these channels are the products of macro-drivers, such as the internationalisation of trade and capital, which are referred in this review as ‘global drivers of ill-health’ [1]. In addition to these aspects of CDoH, the present review discusses ‘unhealthy commodities’, an aspect of CDoH not directly addressed in Kickbush et al.’s framework.

## The commercial determinants of health

Most of the scientific literature on CDoH, as a separate object of inquiry, was published in the past decade and in English, primarily, in high income countries.

### Corporations, unhealthy commodities and the industrial epidemic

NCDs, including cardiovascular diseases and cancers, are the leading cause of mortality globally and responsible for 71% of all deaths [16]. Harmful alcohol drinking, tobacco use, physical inactivity, and the consumption of unhealthy diets, particularly of ultra-processed food products, are the main risks factors for developing NCDs [17]. Other commodities, such as certain chemicals and pesticides, also have a negative impact during their production and use, notably on workers, the health of communities, and the environment [18, 19]. While they might be beneficial, under certain circumstances, the use of motor vehicles [20] and drugs [21], amongst other products, also poses a threat to individuals. These products are collectively described by Stuckler et al. as ‘unhealthy commodities’ [22]. Freudenberg noted that, beyond NCDs, the consumption of unhealthy commodities also leads to a rise in air pollution; a lack of access to essential medicines, to clean water, and to healthy foods; and an increase in the exposure to motor vehicle crashes and gun violence [15]. In 2007, Jahiel and Babor used the concept of an ‘industrial epidemic’, where corporations are described as ‘vectors of diseases’; unhealthy commodities are the agents of these diseases; and individuals the hosts [23]. Jahiel also defined the ‘corporation-induced diseases’ resulting from the industrial epidemic as “diseases of consumers, workers, or community residents who have been exposed to disease agents contained in corporate products” and called for the epidemiological surveillance of these diseases [24]. Collin and Hill further explained that the industrial epidemic is a structural driver of health inequalities [25]. While interventions to prevent and control NCDs and other diseases often target behavioural risks factors, ultimately, it is the exposure to these unhealthy commodities that pose a risk to health [22, 26].

In the literature, the discussion on the impact of corporate activity on public health often focuses on the alcohol, tobacco, and food industries, also known as Big Alcohol [27], Big Tobacco [28], and Big Food [22, 29, 30]. As described above, other industries also have a negative impact on health: the automobile industry [20, 31], the pharmaceutical industry [21], and the mining sector [18], amongst others [32]. In the scientific literature, these corporations are referred to as ‘vectors of diseases’ (or ‘corporate disease vectors’) [23, 33, 34]; ‘industries producing unhealthy commodities’ (and its variant, ‘unhealthy commodity industries’) [22, 26]; ‘dangerous consumption industries’ [35]; and ‘health harming industries’ [36].

### Corporations and practices harmful to health

Corporations seek to make a profit from their commodities. They use ‘business practices’ to run their activities; and ‘market practices’ to develop, produce and sell their commodities [34, 37]. Corporations also use political practices to secure a favourable policy environment [34, 37].

In 2018, Madureira Lima and Galea proposed a framework for understanding the broad impacts that corporate practices have on health [12]. The authors explained that the power of corporations is exerted through five vehicles: political environment; preference shaping; the knowledge environment; the legal environment; and the extra legal environment [12]. The authors later used their framework to evaluate the extent to which corporations have penetrated the “social, political and cultural fabric of a country”, also called ‘corporate permeation’ [38]. In a recent analysis, the authors quantified the corporate permeation in 148 countries [38]. Their evaluation served to better understand the reasons behind the level of consumption of unhealthy commodities, and the existence (or not) of policies to address this consumption in countries [38]. The authors found different levels of corporate permeation across the globe [38].

Baum et al. developed another framework, with a focus on transnationals specifically, called the ‘corporate health impact assessment’ [13]. Their model discusses the political, economic and regulatory environments; the structure, practices and products of corporations; and the health and equity impacts of the activities of corporations [13]. The authors later applied their model for an evaluation of the activities of a fast-food restaurant and a mining company [39, 40]. Their findings showed that corporations could have a positive impact when providing employment in communities, for example, but this could be undermined by the precariousness of such employment, and the negative impacts of the company’s activities on the environment and health [39, 40]. It has been noted that the Baum et al. model, however, fails to address some corporate practices, such as the influence of businesses on science and social norms [12].

For the present review, a distinction was made between the business and the market practices of corporations. The former focuses on the practices that corporations use to run their activities, while the latter focuses on the commodities themselves. Some corporations may market commodities that are considered healthy, such as bananas, but could use business practices that are harmful to health, such as avoiding paying taxes or treating their workers poorly, for example.

Specifically, the business practices of corporations include, amongst other things, the control of the supply chain and market concentration (through mergers and acquisitions for example); labour practices; taxation payments and profits shifting; and the privatisation of utilities [1, 13, 15, 41]. In 2018, Wiist explained that corporate tax avoidance, for example, leads to a shortage of public tax revenue that could be directed to public health purposes and other needs, such as education and housing [42]. There is currently limited literature on these business practices as a separate object of inquiry.

Corporations also use market practices such as product research and development; pricing; marketing, including advertising and retail distribution [1, 15, 25]. There is, for example, ample evidence that the marketing of unhealthy commodities to children and adolescents leads to the increased consumption of these commodities, with associated negative impacts on health [43–45]. Research protocols to systematically study some of these market practices were developed by INFORMAS (International Network for Food and Obesity/non-communicable diseases Research, Monitoring and Action Support) for the food industry [46].

Moreover, corporations use political practices to secure a favourable policy environment [47]. In 2013, Margaret Chan, then Director-General of the World Health Organization, explained: “efforts to prevent non-communicable diseases go against the business interests of powerful economic operators. In my view, this is one of the biggest challenges facing health promotion” [48]. The political influence of corporations is also known as ‘corporate political activity’ (CPA) [47]. The political practices of corporations have been extensively studied, in public health, for the tobacco industry [49–51]. This was facilitated by the access to internal documents in the late 1990s, after litigation against large tobacco transnational companies [52, 53]. These documents have revealed the many ways through which that industry tried to avoid, weaken and delay the development of tobacco control policies [49, 50, 54, 55]. There is growing evidence that other companies in the food [29, 30, 56, 57], alcohol [58–60], gambling [61–63], automobile, and chemicals industries [19, 31, 64], for example, use similar political practices. In public health, these political practices are usually classified into two categories [49]:

- Action-based instrumental strategies:
  - ○ *Coalition management*, for example, when corporations build alliances with third parties, including health organisations, the media and communities [49], or use their so-called ‘corporate social responsibility’ initiatives [65, 66];
  - ○ *Information management*, when corporations try to shape the evidence-base in public health [49]. To that end, they for example commission studies and fund research that would be beneficial to their activities and/or products [49] and/or discredit research that would be supportive of public health policies [49];
  - ○ *Direct involvement and influence in policy*, through their lobbying [49], their provision of financial incentives to policymakers and political parties [49], or their participation in working groups and technical meetings with governments [49], amongst other practices;
  - ○ *Legal strategies* [49];
- Argument-based discursive strategies, where corporations, for example, stress the crucial role that the industry plays in the economy, or promote industry-preferred solutions such as education and voluntary initiatives [49].

It has been argued that corporations not only use these political practices; they often capture different branches of governments [67–69], particularly in market-oriented economies that favour their interests [70]. Some aspects of the Public Health Responsibility Deal in the United Kingdom, a public private initiative that aims to improve public health, have been described in the literature as examples of policy capture [71]. For example, it has been shown that, through the Deal, alcohol industry actors shaped their preferred regulatory regime (co-regulation), while avoiding the introduction of more stringent policies that would have limited harmful alcohol drinking [71].

### Corporations and the global drivers of ill-health

In the literature on CDoH, there is a relatively limited discussion on the global drivers of ill-health, and how these are shaped by, and shaping the practices of corporations [72]. These global drivers indeed influence the business, market, and political practices of corporations, therefore facilitating the manufacture, sale and marketing of unhealthy commodities [1]. Market-driven and neoliberal economies, globalisation, and the development of trade and investment agreements, amongst other factors, have for example contributed to the growing power of corporations in recent decades, but also to the ill-health for the population and planet [22, 41, 72–76]. Transnationals, in particular, control their markets in many countries, some markets being saturated, and others, in low- and middle-income countries, emerging [22, 34, 77–80]. These factors shape the consumption of unhealthy commodities globally. It has also been reported that there is a range of individuals that simultaneously sit on different board of directors of large companies in the alcohol, tobacco, and food industries (among other sectors), thus facilitating the exchange of information between these companies, for an articulated control of markets across the globe [81]. Corporate actors also increasingly shape global health governance, through their philanthropic donations and other interactions with international organisations and governments [82].

## Discussion

**Fig. 1:**
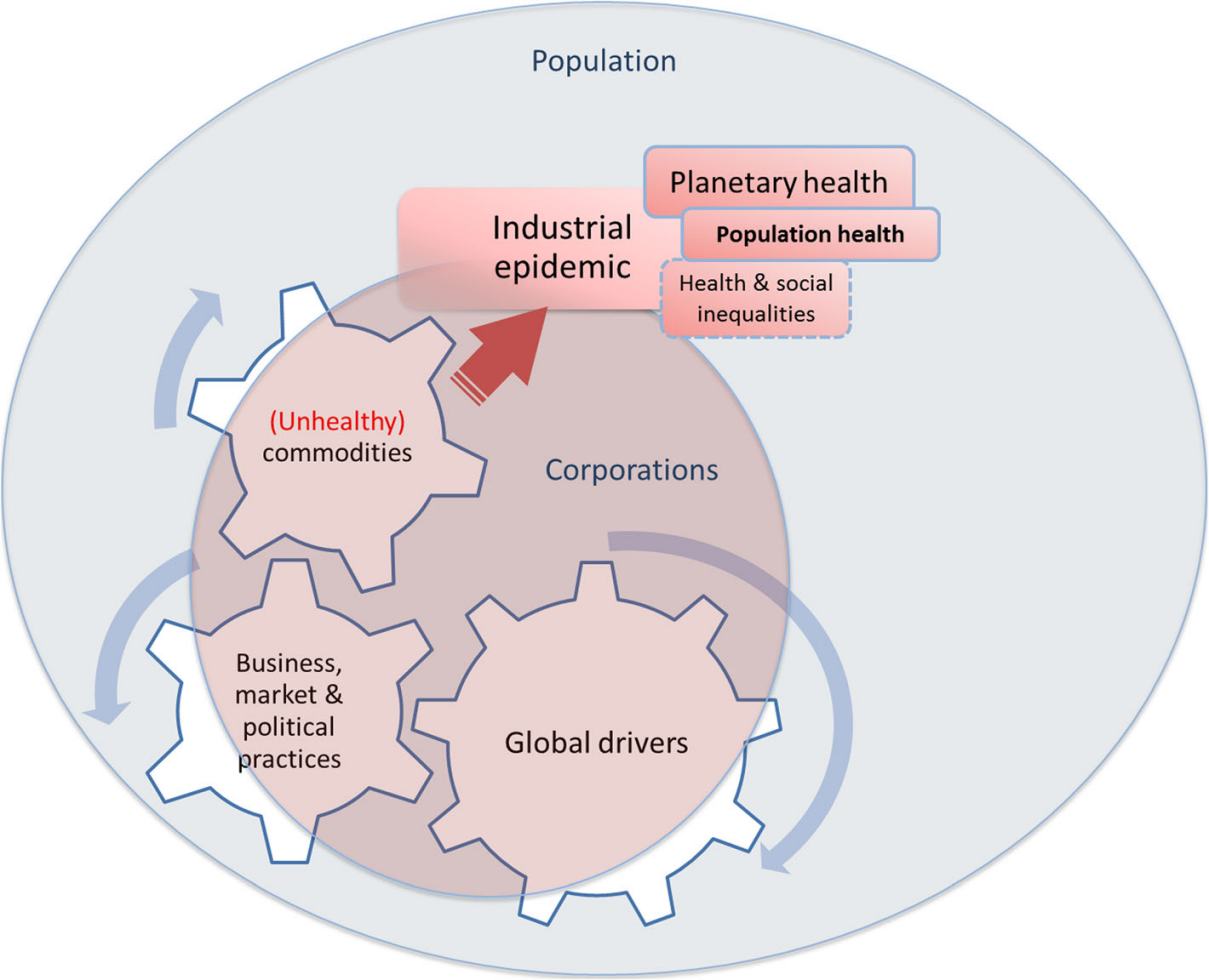
The commercial determinants of health, as discussed in the scientific literature

The present review provides an overview of CDoH, as discussed in the scientific literature, using Kickbush et al.’s framework as a guiding thread. The review therefore covered i) the production of unhealthy commodities by corporations; ii) the use of business, market and political practices that are harmful to health; and iii) global drivers of ill-health, shaped by and shaping the practices of corporations. These different aspects of CDoH have led to an industrial epidemic. Figure 1 presents these different components of CDoH. However, the way these components combine is currently under studied.

Corporations themselves may be affected by CDoH, through the bad health of their workers, which might increase their absence from the workplace. They may also be affected through the bad health of communities in which they operate, which may decrease their purchase power and willingness to buy, and through the damages that they do to the environment, which may affect their business operations, by decreasing their access to natural resources for example.

An exhaustive review of the literature for each of these aspects of CDoH was beyond the scope of the present article. This could be the subject of future studies. There is an emerging literature on corporations and the global drivers of ill-health, and this aspect of CDoH could be studied in future research projects. In addition, the notion of power in relation to CDoH could be further explored. There is, in parallel, a need for more research on the synergies between these different aspects of CDoH, as stressed by Maani et al. [14]. The models developed by Madureira Lima and Galea [12] and Baum and al [13]. could be used for such purposes. Scholars should continue studying each these different aspects of CDoH as drivers of ill-health. Baum et al. proposed a research agenda in this respect [83]. More specifically, McCambridge et al. developed a research agenda for studying the political practices of the alcohol industry, which could be applied across different industries [84]. There is also a need for better synergies in understanding and addressing CDoH not only as drivers of bad health for the population, but also for our planet [76], and as drivers of health and social inequalities. The recent work of the Lancet Commission on the “Global Syndemic of Obesity, Undernutrition and Climate Change” is a step in the right direction, with the questions of power imbalance and corporate practices directly addressed [85]. More research is needed to better evaluate both the negative impacts that corporations have on our health, as described in this review, and the positive impacts their activities have [5, 6].

Finally, scholars recently proposed solutions to address CDoH: challenging corporate power and supporting communities who stand up against harmful corporate practices; introducing regulation that would limit these harmful practices; challenging the corporate narrative in public health [2]. There is currently a need for more research on these solutions. West and Marteau concluded in their commentary on CDoH, in 2013, that “re-thinking macro-economics to achieve prosperity without growth [is] a brave but vital initiative to curb the commercial determinants of health before the planet becomes too hostile to support human existence” [8]. Documenting and learning from successful examples in challenging corporations could be important but addressing the global drivers of ill-health might have more impact if CDoH are to be addressed [15, 76].

## Conclusion

There is currently limited research on CDoH as a separate object of study and a lack of attention, in particular, to the global drivers of ill-health. There is also limited research on the activities of industries other than the food, alcohol and tobacco industries. The discussion on CDoH offers a unique opportunity to shift the dominant paradigm in public health, where individual behaviours are considered to be driven by inadequate environments. Ill-health, damages to the environment, and health and social inequalities, could rather be understood as shaped by CDoH.

## Data Availability

All data generated or analysed during this study are included in this published article.
